# Analgesic effect of apricot kernel oil on neuropathic pain in rats

**DOI:** 10.1016/j.heliyon.2024.e34988

**Published:** 2024-07-20

**Authors:** Maryam Akaberi, Fatemeh Forouzanfar, Hassan Rakhshandeh, Seyed Mostafa Moshirian-Farahi

**Affiliations:** a Department of Pharmacognosy, School of Pharmacy, Mashhad University of Medical Sciences, Mashhad, Iran; b Medical Toxicology Research Center, School of Medicine, Mashhad University of Medical Sciences, Mashhad, Iran; c Pharmacological Research Center of Medicinal Plants, Mashhad University of Medical Sciences, Mashhad, Iran

**Keywords:** Neuropathic pain, Inflammation, Oxidative stress, Apricot kernel oil, Herbal medicine

## Abstract

**Background:**

A somatosensory nerve lesion or disease causes neuropathic pain. Presently, prescribed treatments are unsatisfactory or ineffective. The kernel oil of the apricot tree (*Prunus armeniaca* L) is known for its anti-inflammatory and antioxidant effects. This study investigated the effect of apricot kernel oil in chronic constriction injury (CCI)- induced neuropathic pain in rats.

**Materials/Methods:**

Liquid chromatography-electrospray mass spectrometry (LC-ESIMS) analysis was carried out to gain a deeper understanding of the apricot kernel oil's main compounds. Rats were treated daily with apricot kernel oil (2 and 4 ml/kg) or gabapentin (100 mg/kg) for 14 days after CCI induction. Hot plate, acetone drop, and Von Frey hair tests were performed to evaluate thermal and mechanical activity. Spinal cord malondialdehyde (MDA), total thiol, interleukin (IL)-1β, and tumor necrosis factor α (TNF-α) levels were assessed to measure biochemical changes.

**Results:**

The most detected compounds in apricot kernel oil were lipids and fatty acids. CCI produced a significant increase in thermal hyperalgesia, mechanical allodynia, and cold allodynia. Moreover, CCI increased the inflammation and oxidative stress markers in spinal cord samples. Oral administration of apricot kernel oil and gabapentin significantly decreased the CCI-induced nociceptive pain threshold. Besides, spinal cord biochemical changes were attenuated.

**Conclusions:**

Our findings suggest that apricot kernel oil could attenuate neuropathic pain, possibly through anti-inflammatory and antioxidant properties.

## 1 Introduction

Pain is the response of the living body to actual or potential tissue damage. Pain brings a distressing sensory and emotional experience [1]. Neuropathic pain caused by lesions in the peripheral nervous system is classified in the chronic pain category as particularly intense and hard to treat [2]. Allodynia, hyperalgesia, and abnormal pain are important symptoms of neuropathic pain [3]. Oxidative stress is one of the main contributing factors in the initiation and development of neuropathic pain. Increased reactive oxygen species (ROS) cause central sensitization by activating the second messengers in the dorsal horn and glial cells in the spinal cord [4,5]. The weak endogenous antioxidant system of nerve cells makes them more susceptible to nerve dysfunction after nerve injury [6]. Immune cells at the site of injury, such as mast cells, macrophages, and glial cells, become activated upon nerve damage and release proinflammatory mediators, including interleukin (IL)-1β, interferon-gamma (IFN-y), IL-6, and tumor necrosis factor α (TNF-α). These proinflammatory mediators can lower the threshold of nociceptive perception and drive the progression of neuropathic pain [7]. Patients with neuropathic pain have a worse health-related quality of life [8]. Moreover, analgesics are associated with adverse effects, and patients become more resistant over time [8]. Because neuropathic pain is a challenging condition to treat, the search for novel analgesics is still ongoing. There has been a great deal of interest in plants as a source of drug discovery, even in the modern era [9,10]. The apricot (*Prunus armeniaca* L.) is from the family *Rosaceae*. It is mainly cultivated in China, the central Asian center (from Tien-Shan to Kashmir), or the near-eastern center (Iran, Caucasus, and Turkey) [11]. The characteristics of apricot fruit such as variety, origin, maturity state, and climate condition, affect the nutrients and composition of apricot oil [12]. There are several biologically active compounds in apricot kernel oil, such as phenols, carotenoids, anthocyanins, flavonoids, vitamin E, and quality proteins [12]. Apricot kernel oil also has several pharmacological effects, such as anti-cancer [13], cardio-protective [14], and gastro-protective [15]. Given the existing literature on apricot kernel oil's antioxidant [16] and anti-inflammatory [17] effects, our study was conducted to examine the effect of apricot kernel oil on neuropathic pain using a chronic constriction injury (CCI) rat model.

## 2 Materials and methods

### 2.1 Extraction of apricot kernel oil

The apricot kernels were purchased from an herbal store (Mashhad, Iran). The seeds were peeled, cleaned, dried, and broken into several pieces. Then, the oil of the apricot kernel was obtained using the cold press method [18]**.**

### 2.2 Liquid chromatography-electrospray mass spectrometry (LC-ESIMS) analysis

LC-ESIMS analysis was carried out to gain a deeper understanding of the apricot kernel oil's metabolic profile. The substances in the sample were identified by ESIMS characteristics and separated using an improved chromatography method (LC technique). The sample's concentration of dimethyl sulfoxide (DMSO) was 10 mg/mL. For the separation, a SUPELCO analytical Discovery HS C18 column (150 mm × 4.6 mm, 3 μm) was utilized. The requirement for separation was: Water and 0.1 % formic acid makeup Solvent A, whereas acetonitrile and 0.1 % formic acid make up Solvent B. Gradient profile: 40 min for 5%–95 % solvent B; 40–70 min for 95 % solvent B.

The sample injection volume was 20 μL, the column temperature was 30 °C, and the solvent flow rate was 0.25 ml/min. The mode of negative ionization was used to gather the mass data. There was a 4.5 kV capillary voltage. The gas nebulizer (grade 5) was nitrogen (N2). 450 °C was the desolvation temperature. Mass Convert software (ProteoWizard) was used to convert the raw MS data into MzXML files, which were then processed using MZmine 3.0. The parameters were set according to our previous study [19].

### 2.3 Animals

Adult male Wistar rats (220–270 g) were provided by the Mashhad University of Medical Sciences (MUMS), animal experimental center, in Mashhad, Iran. The rats were maintained in laboratory conditions with free access to water and food.

### 2.4 CCI-induced neuropathic pain model

According to the previous report, a rat model of CCI-induced neuropathic pain was established [20]. The rats were anesthetized intraperitoneally with a ketamine-xylazine solution (100, and 10 mg/kg, respectively). The right common sciatic nerve was exposed via a lateral approach, and four chromic gut ligatures (4–0) were placed loosely around it with a 1 mm interval between each.

### 2.5 Experimental design

Five groups of eight animals were assigned after the animals had been acclimated:

1 Control group (the sciatic nerve was exposed, but not ligated)
2 CCI model group.
3 CCI animals treated with apricot kernel oil (2 ml/kg) by gavage for 14 days.
4 CCI animals treated with apricot kernel oil (4 ml/kg) by gavage for 14 days.
5 CCI animals treated with gabapentin (100 mg/kg) by gavage for 14 days.

A 0.9 % saline solution was given to the control rats and the rats that underwent CCI surgery.

Oral administration of the apricot kernel oil, or gabapentin, was begun and continued until 14 days after the CCI surgery.

Previous studies were used to obtain the dose of apricot kernel oil for this study [14,21,22].

### 2.6 Behavioral tests

#### 2.6.1 Assessment of mechanical allodynia

Mechanical sensitivity was evaluated with Von Frey's filaments. In summary, rats were placed in the testing room for 15 min for them to acclimate to the new environment and become calm. The Von Frey filaments were 0.6, 1.0, 1.4, 2.0, 4.0, 6.0, 8.0, 10.0, 15.0, 26.0, and 60-g forces. On the plantar surface of a rat paw, Von Frey stimuli were applied to it, and the force with which it was elicited to withdraw the paw was recorded. A response to at least three out of five times resulted in those withdrawal attempts being recorded as the paw withdrawal threshold. The cut-off was 60 g [23].

#### 2.6.2 Assessment of cold allodynia

A chamber made of Plexiglas was placed in which rats were placed and acclimated for 15 min at a time. Cold allodynia was determined as the number of foot withdrawal responses after applying an acetone bubble to the injured hind paw of the animal. The procedure was repeated five times (once every 5 min) for a total of five times. Thermal withdrawal frequency is determined using the following formula: (number of trials with foot withdrawal) × (total number of trials)/100 [24].

#### 2.6.3 Assessment of heat hyperalgesia

To evaluate heat hyperalgesia, the rats were placed on top of a hot plate set to 50 °C. The response latency to either a left hind-paw lick or jump was recorded. The maximum time was 15 s to prevent tissue damage [25].

#### 2.6.4 Biochemical assessment

After completing the behavioral experiments on day 14, animals were deeply anesthetized with xylazine (10 mg/kg, i.p.) and ketamine (100 mg/kg, i.p.), and the L4-L6 section of the spinal cord were separated to perform biochemical assays. The determination of IL-1β and TNF-α in serum samples was performed according to the manufacturer's instructions for commercial ELISA kits (Karmania Pars Gene Company, Kerman, Iran).

The L4/6 spinal cord samples were homogenized. The total thiol level was assayed by Ellman's spectrophotometric method [26]. First, 50 μl of homogenate and 1 ml of Tris-EDTA buffer (pH 8.6) were mixed, and the absorbance was read against Tris-EDTA buffer alone (R1). Then, 20 μL of 10 mM DTNB (5,5′-dithiobis-(2-nitrobenzoic acid) solution was added to the mixture. This mixture was kept at room temperature for 15 min, and the absorbance of the sample (R2) was read again. The absorbance of the DTNB reagent (B) was also read. (absorbances were read at 412 nm). The total thiol concentration was calculated as follows: (micromole/gr tissue) = (R2 – R1 – B) × 1.07/0.05 × 13.6 [27]. For MDA measurement; 1 ml of homogenate was mixed with 2 ml of Trichloroacetic acid (TCA)- thiobarbituric acid (TBA)-HCl reagent and boiled for 40 min. After the mixture reached room temperature, the mixture was centrifuged at 3000 rpm for 10 min. The supernatant was collected and read at 532 nm against the reagent blank. The MDA level was determined using the following formula: 1.56 × 10^5^ M^−1^ cm^−1^ and expressed in nmol/g tissue [27].

### 2.7 Statistics

Results are expressed as the mean ± standard error of the mean (SEM). Two-way ANOVA, one-way ANOVA, and Tukey's post hoc tests were utilized to estimate the differences in behavioral and biochemical data. P < 0.05 was considered statistically significant.

## 3 Results

**Fig. 1:**
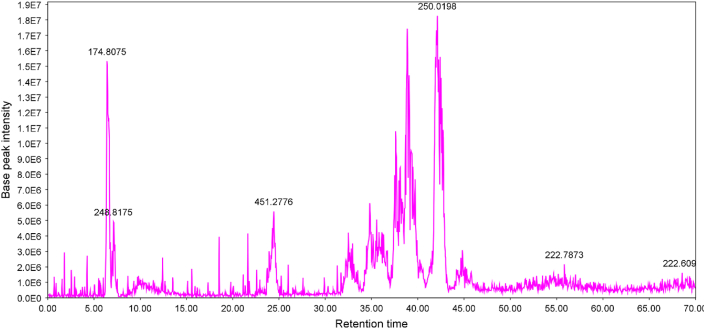
LC-ESIMS chromatogram of the apricot kernel oil.

Fig. 1 shows the LC-MS chromatogram of apricot kernel oil, and Table 1 represents the tentatively identified components of the oil. As it is obvious from the table and according to the LC-MS data, the most detected compounds might be lipids and fatty acids.

**Table 1:** The tentatively identified compounds in the apricot kernel oil (negative ionization mode).

No.	Retention time	[M-H^+^]^-^	Tandem mass spectrometry	Tentative identity	References
1	24.49	451.2045	225.0250, 433.8500 [M-H_2_O-H^+^]^-^, 487.0286, 565.3143	An acylglycerol	[28]
2	32.50	329.0613	201.0850 [M-H_2_O-H^+^]^-^, 310.8167	Unresolved	–
3	34.86	329.3297	199.0354, 311.6348	Unresolved	–
4	37.71	281.5875	200.8347	Oleic acid or Octadecenoic acid	[28]
5	38.86	279.0089	–	Caprylic acid	[28,29]
6	38.87	315.1488	170.8111, 296.8755 [M-H_2_O-H^+^]^-^	Unresolved	–
7	39.82	279.4000	233.3133	Linoleic acid	[28]
8	42.12	267.0000	249.9411 [M-H_2_O-H^+^]^-^314.7872	Methyl palmitoleate	[28,29]
9	43.77	281.0200	–	Oleic acid or Octadecenoic acid	[28]

### 3.1 Behavioral assessment results

A pre-surgery test conducted on day 0 (1 day before surgery) showed that there were no significant differences between the animals in their responses to von Frey filaments.

After CCI, the mean PWT in the right hind paw decreased on days 3–14, in comparison to the control rats (p < 0.001). In CCI rats treated with 4 ml/kg of apricot kernel oil once a day, tactile allodynia was partially attenuated and measured on days 3, 5, 7, 10, and 14 post-surgery (p < 0.01, p < 0.001, p < 0.001, p < 0.001 and p < 0.001, respectively).

**Fig. 2:**
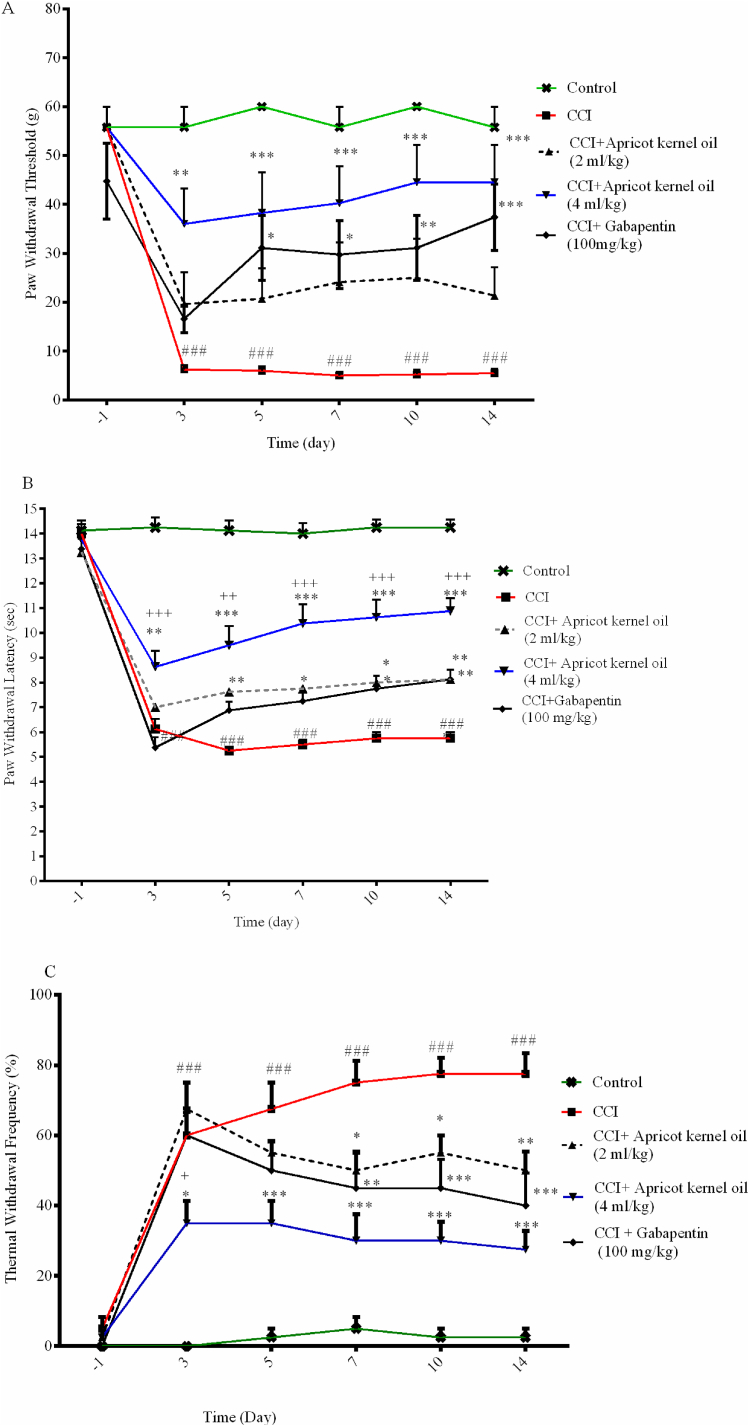
Effect of apricot kernel oil on (A) mechanical allodynia, (B) heat hyperalgesia (C), and cold allodynia in CCI-induced neuropathic pain in rats. Each point represents the mean ± SEM (n = 8). ###p < 0.001 significantly different from the control group. *p < 0.05, **p < 0.01, and ***p < 0.001, significantly different from the CCI group. ^+^p < 0.05, ^++^p < 0.01, and ^+++^p < 0.001 significantly different from the gabapentin group.

PWT was significantly attenuated in 100 mg/kg of gabapentin, compared to the control-operated rats on 5, 7, 10, and 14 post-operations (p < 0.05, p < 0.05, p < 0.01, andp <0.001, respectively (Fig. 2A).

The results of the baseline PWL evaluation, performed one day before surgery (day −1), showed no significant variation from group to group and were relatively stable. CCI rats showed significantly lower PWL versus the control-operated rats at 3–14 days post CCI surgery (p < 0.001).

Apricot kernel oil at doses of 4 ml/kg on days 3, 5, 7, 10, and 14 after operation increased the PWL on the hind paw affected CCI in a significant manner (p < 0.01, p < 0.001, p < 0.001, p < 0.001, and p < 0.001 respectively). Apricot kernel oil at doses of 2 ml/kg on days 5, 7, 10, and 14 after operation increased the PWL on the hind paw and affected CCI in a significant manner (p < 0.01, p < 0.05, p < 0.05 and p < 0.01, respectively). Gabapentin on days 10 and 14 after the operation increased the PWL on the hind paw and affected CCI in a significant manner (p < 0.05, and p < 0.01, respectively). PWL significantly increased in the group receiving apricot kernel oil at doses of 4 ml/kg on days 3, 5, 7, 10, and 14 after operation compared to gabapentin treated group (p < 0.001, p < 0.01, p < 0.001, p < 0.001, and p < 0.001 respectively). (Fig. 2B).

The CCI rats showed a significant increase in TWF on days 3–14 versus the control group (p < 0.001). However, apricot kernel oil (2 ml/kg) decreased TWF on days 7, 10, and 14 versus the CCI-control group (p < 0.05, p < 0.05, and p < 0.01, respectively). Gabapentin (100 mg/kg) decreased TWF on days 7, 10, and 14 versus the normal saline-treated CCI animals (p < 0.01, p < 0.001, and p < 0.001, respectively). Apricot kernel oil, at 4 ml/kg, resulted in a significant decrease in TWF on days 3, 5, 7, 10, and 14 versus the normal saline-treated CCI animals (p < 0.05, p < 0.001, p < 0.001, p < 0.001, and p < 0.001, respectively) (Fig. 2C). TWF significantly decreased in the group receiving apricot kernel oil at doses of 4 ml/kg on days 3 after operation compared to the gabapentin-treated group (p < 0.05), (Fig. 2C).

### 3.2 Biochemical results

**Fig. 3:**
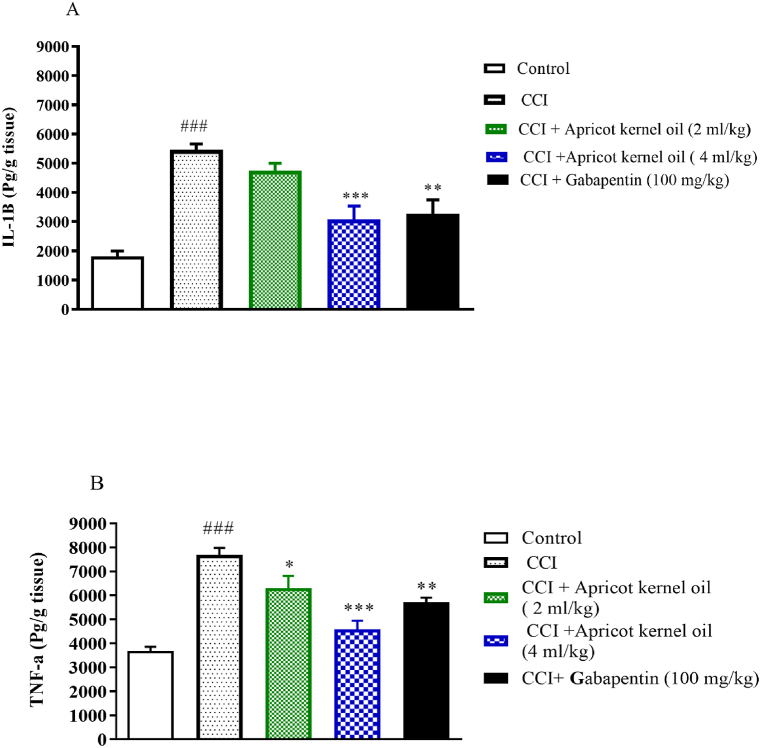
Effect of apricot kernel oil on (A) IL-1β (B) TNF-α levels in CCI-induced neuropathic pain in rats. Each point represents the mean ± SEM (n = 8). ###p < 0.001 significantly different from the control group. *p < 0.05, **p < 0.01, and ***p < 0.001, significantly different from the CCI group.

The results showed that the level of IL-1β in the CCI group was significantly higher versus the control group in spinal cord samples (P < 0.001, Fig. 3A). Apricot kernel oil, at 4 ml/kg, and gabapentin resulted in significant decreases of IL-1β; p < 0.001, and p < 0.01 respectively (Fig. 3A). The level of TNF-α in the spinal cord samples was significantly higher in the normal saline-treated CCI animals versus the control-operated rats (P < 0.001, Fig. 3B).

Apricot kernel oil, at 2 ml/kg, 4 ml/kg, and gabapentin resulted in significant decreases in the levels of TNF-α (P < 0.05, P < 0.001, and P < 0.01) versus the normal saline-treated CCI animals (Fig. 3B).

**Fig. 4:**
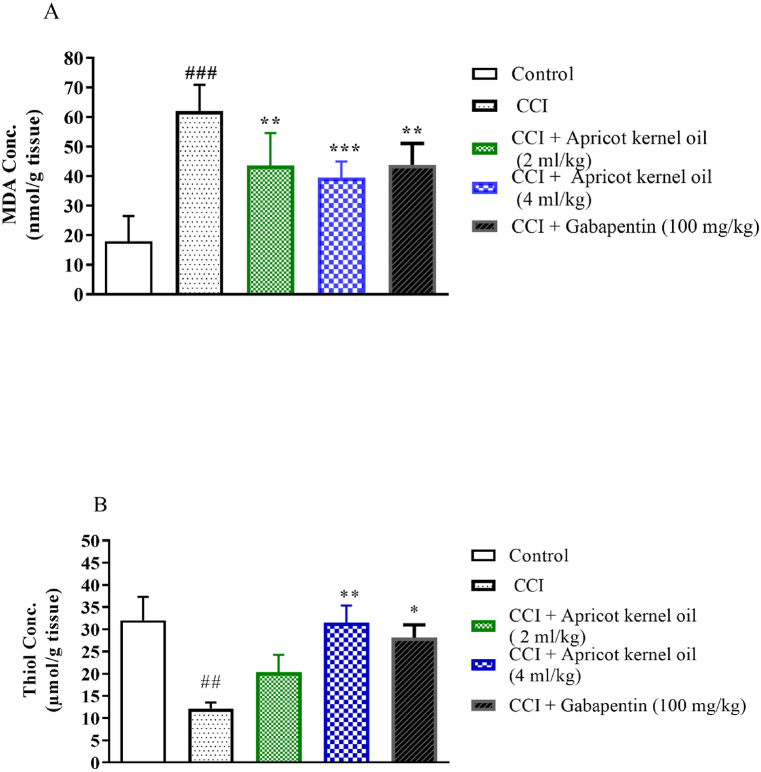
Effect of apricot kernel oil on (A) MDA (B) thiol levels in CCI-induced neuropathic pain in rats. Each point represents the mean ± SEM (n = 8). ###p < 0.001 significantly different from the control group. *p < 0.05, **p < 0.01, and ***p < 0.001, significantly different from the CCI group.

**Fig. 5:**
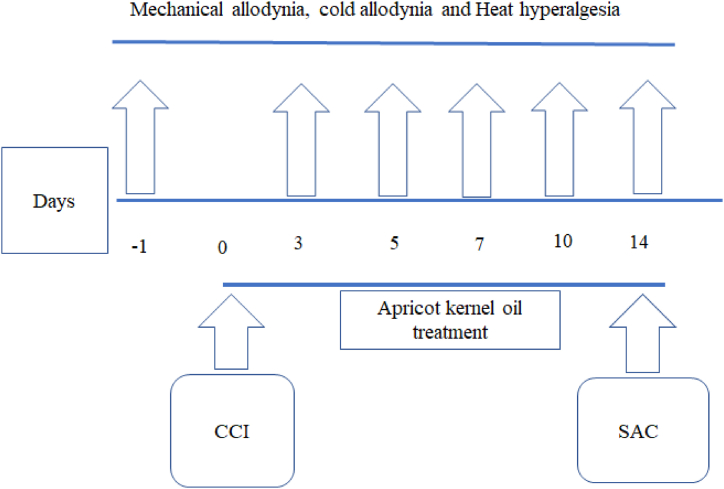
Diagrammatic sketch for the behavioral, and biochemical experiments. CCI: chronic constriction injury; SAC: sacrificed for biochemical experiments. Day 0 refers to the day of surgery.

The level of MDA in the spinal cord was significantly higher in the normal saline-treated CCI animals (CCI group) versus the control-operated rats (P < 0.001, Fig. 4A). Apricot kernel oil, at 2 ml/kg, 4 ml/kg, and gabapentin resulted in significant decreases in the MDA (P < 0.01, P < 0.001, and P < 0.01) versus the CCI group (Fig. 4A). The level of thiol was significantly lower in the normal saline-treated CCI animals versus the control-operated rats (P < 0.01, Fig. 4B). Apricot kernel oil, at 4 ml/kg, and gabapentin resulted in significant increases in total thiol levels versus the CCI group (P < 0.01, P < 0.05, respectively) (Fig. 4B). Fig. 5 showes the diagrammatic sketch for the behavioral, and biochemical experiments.

## 4 Discussion

In this study, our behavioral experiments showed that apricot kernel oil treatment increases PWT and PWL while decreasing TWF. The results of biochemical tests showed that apricot kernel oil reduces inflammation and oxidative stress.

The apricot kernel has about 50 % oil. Apricot kernel oil is rich in triacylglycerols (98 %), followed by phospholipids (1.1 %) and free fatty acids (0.2 %). Triacylglycerols containing three oleic acids (35–42 %), linoleic acid and two oleic acids (22–28 %), two linoleic acids and an oleic acid (7–16 %), a linoleic acid, an oleic acid and a palmitic acid, two linoleic acids and a stearic acid (6–7%), and two oleic acids and a palmitic acid (6–10.4 %) are the major triglyceride content of apricot kernel oil. Unsaturated fatty acids, such as oleic acid (18:1) and linoleic acid (18:2), constitute the most reported fatty acids in apricot kernel oil [30].

Oleic acid, a mono-unsaturated fatty acid, has anti-inflammatory effects [31]. Linoleic acid, a polyunsaturated fatty acid, has beneficial effects on oxidative stress and inflammatory responses [32]. Palmitic acid is the most abundant saturated fatty acid in apricot kernel oil.

In addition to the above-mentioned components, apricot kernel oil is a good source of phytosterols, tocochromanols, carotenoids, and polyphenols. Δ7-stigmasterol, β-Sitosterol, campesterol, cholesterol, 24-methylene–cycloartanol, gramisterol, Δ5-avenasterol, Δ7-avenasterol, and citrostadienol are the most important phytosterols. δ-tocopherol, α-tocopherol, and γ-Tocopherol are the major tocochromanols. β-cryptoxanthin, zeaxanthin, Lutein, and β-carotene constitute the most reported carotenoids in apricot kernel oil. Apricot kernel oil, with its polyphenols, leads to the elimination of free radicals in the body and thus protects it against oxidative stress. Moreover, apricot kernel oil also contains volatile compounds such as furfural, benzaldehyde, 2-methyl-propanal, 2,5-dimethyl-pyrazine, methoxy pyrazine, 2-methyl-butyl aldehyde, nonanal, methylpyrazine, and 3-ethyl-2,5-dimethyl-pyrazine. However, the major volatile constituent is benzaldehyde [30]. Phytochemicals such as carotenoids, and polyphenolic compounds showed antinociceptive effects in animal models of neuropathic pain [33,34].

Chronic neuropathic pain, which is characterized by an abnormally increased sensitivity to pain.

(hyperalgesia), the perception of harmless stimuli as painful (allodynia), and spontaneous pain, can be caused by damage to the nervous system. The brain and spinal cord's processing neurons undergo secondary alterations due to the hyperactivity of nociceptors, which causes mechanoreceptive A-fiber input to be interpreted as pain. Additional hyperexcitability may result from neuroplasticity alterations in the central pain modulatory systems. The sensitization of primary afferent nociceptors is caused by molecular mechanisms that include growth factor release from degenerating nerve fibers, upregulation of voltage-gated sodium channels, and different types of receptor proteins. There is evidence of sensitization of brain neurons as well as significant secondary alterations in the spinal cord dorsal horn due to peripheral nociceptor hyperactivity [35].

We chose the CCI model for the surgery of the sciatic nerve and the establishment of neuropathic pain because it is a commonly accepted model for producing reliable resemblances between clinical manifestations of neuropathic pain [36]. The CCI model in our rats successfully exhibited allodynia and hyperalgesia. Apricot kernel oil and gabapentin reduced the allodynia and hyperalgesia at different times of the experiment.

Gabapentin is an anti-epileptic medication but it is used as a first-line therapy in the treatment of neuropathic pain [37]. In our study gabapentin treatment reduced the pain behavior, oxidative stress, and inflammation in neuropathic rats.

It has been shown that proinflammatory and inflammatory cytokines like IL-1β, interferon-gamma (IFN-y), IL-6, and TNF-α can increase spinal neuroinflammatory and immunological activity. It is these events that greatly contribute to the initiation and development of neuropathic pain after a nerve injury [[38], [39], [40]]. In confirmation, studies have demonstrated that reducing the TNF-α, and IL-1β produces pain-relieving effects [41,42].

In response to nerve injuries, mast cells, macrophages, and glial cells release proinflammatory mediators such as IL-1β, IFN-y, IL-6, and TNF-α. In addition, proinflammatory cytokines as well as neurotoxic products are produced and released from glial cells located in the spinal cord. The inflammatory processes at the site of injury lower the threshold of nociceptive perception and drive the progression of neuropathic pain [7]. The generation of pro-inflammatory cytokines is additionally affected by ROS production by polymorphonuclear neutrophils [43].

Also, Increased TNF-α, which is a pro-inflammatory cytokine, stimulates the release of other inflammatory cytokines [44]. Likewise, we found increased TNF-α and IL-1β concentrations after CCI, which demonstrated neuroinflammation. Also, our results showed that TNF-a and IL-1β were decreased following apricot kernel oil administration in CCI rats in the spinal cord.

A situation of oxidative stress occurs when there is an increase in the ratio between free radicals and antioxidants [45]. The most abundant form of free radicals is ROS. The mitochondrial metabolism generates several ROS, such as superoxide anions, hydrogen peroxide, and hydroxyl radicals, as a result of the metabolism. Cellular mechanisms can be damaged by ROS, and proteins and lipids can be oxidized. ROS can also damage DNA, resulting in apoptosis and cell death, along with previous pathologies [45,46].

Oxidative stress is a key pathophysiological factor in peripheral neuropathy. ROS causes central sensitization through the activation of second messengers in the dorsal horn cells and the activation of spinal glial cells [4,5]. Nervous tissues have weak endogenous antioxidant defenses, making them more prone to neuronal damage [6]. Increased lipid peroxidation after nerve injury can be demonstrated by increased MDA [47]. Sulfhydryl groups (also called thiol groups) act as cofactors for several enzymes that act as antioxidants [48]. In the present study, MDA levels were higher and thiol levels were lower in the spinal cord of CCI rats compared to control rats. We found that apricot kernel oil enhanced thiol levels while reducing MDA levels in the spinal cord samples. Previous studies have demonstrated that apricot kernels exert antioxidative, anti-inflammatory, and other beneficial effects. It has been found that apricot kernel oil decreased gastric mucosal damage and ulcer index in rats with ethanol-induced gastric mucosal injury. Catalase (CAT) and superoxide dismutase (SOD) activities in the gastric mucosa were also increased by apricot kernel oil, and MDA concentration was significantly decreased by it. A significant decrease in IL-6 and an increase in IL-10 levels were also observed in gastric tissue following treatment with apricot kernel oil [15].

In hypercholesteremic rats supplemented with apricot oil, oxidative stress status was assessed. The liver of hypercholesteremic rats had decreased CAT and glutathione peroxidase (GPx) enzyme levels, which were improved upon treatment with apricot kernel oil [49]. In one study administration of apricot kernel oil increased CAT, GPx, and SOD myocardial activities, whereas decreased MDA level in myocardial ischemia–reperfusion in a rat model [14]. Another study showed that dried apricot administration led to an increase in CAT, glutathione (GSH), and SOD and decrement in MDA levels in methotrexate-induced oxidative damage in rat kidneys. Besides following treatment with apricot glomerulosclerosis and apoptosis decreased in kidney tissue [50]. Pretreatment with ethanolic apricot seed extracts reduced the levels of liver enzymes (ALP, ALT, and AST). Besides, it reduced oxidative stress as demonstrated by the reduction of MDA and the increment of GSH levels in N-nitrosodiethylamine -induced hepatocarcinogenesis in rats [51]. Administration of ground apricot kernel increased liver CAT and SOD activities, whereas it decreased the MDA level and liver fibrosis induced by dimethylnitrosamine in rats [52].

Feeding with 5 % bitter apricot kernels was found to reduce the area of liver injury. After CCl4 was administered, there was an increase in serum AST, ALT, TOS activity, liver Bcl 2, and NFƙB levels. However, adding the bitter apricot kernel to their diet significantly decreased their activity levels. The administration of bitter apricot kernel increased the levels of serum TAS as well as hepatic Bax, caspase 3, and Nrf2 compared to the CCl4 group. Massive necrosis in the centrilobular region was found by histopathological analysis, and dietary supplementation with bitter apricot kernel concentrates was found to mitigate the degenerative alterations brought on by CCl4 [53]**.**

Significant analgesic and anti-inflammatory properties were demonstrated by the ethanolic extracts of apricot seeds, in formalin-induced paw edema, writhing, and hot plate tests [54].

## 5 Conclusion

In conclusion, our results demonstrated that treatment with apricot kernel oil provides significant analgesic properties by reducing hyperalgesia and allodynia in neuropathic rats. Apricot kernel oil could prevent lipid peroxidation and restore thiol levels in neuropathic rats. In addition, it was demonstrated that it was able to reduce inflammation, which is important in the pathology of neuropathic pain. The analgesic effect of apricot kernel oil needs to be tested in other pain models to clarify the exact mechanism.

## Funding

The authors gratefully acknowledge the financial support from the Vice-Chancellor for Research and Technology, 10.13039/100019155MUMS, Mashhad, Iran (990928)**.**

## Data availability statement

Data can be available upon request from the corresponding author.

## Ethics approval

All animal experiments were approved by Mashhad University of Medical Sciences, Ethics Committee Acts (990928; ethics approval code: IR.MUMS.MEDICAL.REC.1400.336)**.**

## CRediT authorship contribution statement

**Maryam Akaberi:** Writing – original draft, Investigation, Formal analysis. **Fatemeh Forouzanfar:** Writing – review & editing, Writing – original draft, Supervision, Investigation, Formal analysis. **Hassan Rakhshandeh:** Investigation. **Seyed Mostafa Moshirian-Farahi:** Investigation.

## Declaration of competing interest

The authors declare that they have no known competing financial interests or personal relationships that could have appeared to influence the work reported in this paper.

## References

[1] Raja S.N. (2020). The revised IASP definition of pain: concepts, challenges, and compromises.

[2] Asgharzade S. (2020). A review on stem cell therapy for neuropathic pain. Curr. Stem Cell Res. Ther..

[3] Meacham K. (2017). Neuropathic pain: central vs. peripheral mechanisms. Curr. Pain Headache Rep..

[4] Zhang X. (2003). The effects of protein phosphatase inhibitors on nociceptive behavioral responses of rats following intradermal injection of capsaicin.

[5] Raghavendra V. (2003). Anti-hyperalgesic and morphine-sparing actions of propentofylline following peripheral nerve injury in rats: mechanistic implications of spinal glia and proinflammatory cytokines.

[6] Lee K.H., Cha M., Lee B.H. (2020). Neuroprotective effect of antioxidants in the brain. Int. J. Mol. Sci..

[7] Pinho-Ribeiro F.A., Verri W.A., Chiu I.M. (2017). Nociceptor sensory neuron-immune interactions in pain and inflammation. Trends Immunol..

[8] Attal N. (2011). The specific disease burden of neuropathic pain: results of a French nationwide survey. Pain.

[9] Pourbagher‐Shahri A.M., Forouzanfar F. (2023). Saffron (Crocus sativus) and its constituents for pain management: a review of current evidence. Phytother Res..

[10] Rakhshandeh H. (2022). Effects of Capparis Spinosa extract on the neuropathic pain induced by chronic constriction injury in rats. Metab. Brain Dis..

[11] Roussos P.A. (2016). Nutritional Composition of Fruit Cultivars.

[12] Akhone M.A. (2022). Apricot kernel: bioactivity, characterization, applications, and health attributes. Foods.

[13] Etminan A. (2022). Ultrasonic nano emulsification of apricot kernel oil and its therapeutics effects on suppression of human lung cancer cells (A549). Mater. Technol..

[14] Zhang J. (2011). Protective effects of apricot kernel oil on myocardium against ischemia–reperfusion injury in rats. Food Chem. Toxicol..

[15] Karaboğa İ. (2018). Gastroprotective effect of apricot kernel oil in ethanol-induced gastric mucosal injury in rats. Biotech. Histochem..

[16] Makrygiannis I. (2023). Exploring the chemical composition and antioxidant properties of apricot kernel oil. Separations.

[17] Minaiyan M. (2014). Anti-inflammatory effect of Prunus armeniaca L.(Apricot) extracts ameliorates TNBS-induced ulcerative colitis in rats. Research in Pharmaceutical Sciences.

[18] Hao Y. (2022). A comparative study of apricot kernel oil yield using different extraction methods. Bioresources.

[19] Zanganeh, F., et al., Dereplication of natural cytotoxic products from Helichrysum oligocephalum using ultra‐performance liquid chromatography–quadrupole time of flight‐mass spectrometry*.* Separ. Sci.: p. 2300150.

[20] Bennett G.J., Xie Y.-K. (1988). A peripheral mononeuropathy in rat that produces disorders of pain sensation like those seen in man. Pain.

[21] Ilias B. (2022). Protective effects of apricot oil against mercuric chloride-induced hepato-renal toxicity in rats. Egyptian Academic Journal of Biological Sciences, D. Histology & Histochemistry.

[22] Tian H. (2016). Apricot kernel oil ameliorates cyclophosphamide‐associated immunosuppression in rats. Lipids.

[23] Forouzanfar F. (2023). Cerium oxide nanoparticles ameliorate oxidative stress, inflammation, and pain behavior in neuropathic rats. Curr. Neurovascular Res..

[24] Rakhshandeh H. (2021). Pain-relieving effects of Lawsonia inermis on neuropathic pain induced by chronic constriction injury. Metab. Brain Dis..

[25] Kukkar A., Singh N., Jaggi A.S. (2013). Neuropathic pain-attenuating potential of aliskiren in chronic constriction injury model in rats. J. Renin-Angiotensin-Aldosterone Syst. JRAAS.

[26] Ellman G.L. (1959). Tissue sulfhydryl groups. Arch. Biochem. Biophys..

[27] Forouzanfar F. (2019). Attenuating effect of Portulaca oleracea extract on chronic constriction injury induced neuropathic pain in rats: an evidence of anti-oxidative and anti-inflammatory effects.

[28] Hrichi S. (2020). Identification of fatty acid, lipid and polyphenol compounds from Prunus armeniaca L. Kernel extracts. Foods.

[29] Makrygiannis I. (2023). Exploring the chemical composition and antioxidant properties of apricot kernel oil. Separations.

[30] Kiralan M., Ramadan M.F. (2019). Fruit Oils: Chemistry and Functionality.

[31] Santa-María C. (2023). Update on anti-inflammatory molecular mechanisms induced by oleic acid. Nutrients.

[32] Saha S.S., Ghosh M. (2012). Antioxidant and anti-inflammatory effect of conjugated linolenic acid isomers against streptozotocin-induced diabetes. Br. J. Nutr..

[33] Boadas-Vaello P., Miguel Vela J., Verdu E. (2017). New pharmacological approaches using polyphenols on the physiopathology of neuropathic pain. Curr. Drug Targets.

[34] Hernández-Ortega M. (2012). Antioxidant, antinociceptive, and anti-inflammatory effects of carotenoids extracted from dried pepper (Capsicum annuum L.). J. Biomed. Biotechnol..

[35] Baron R. (2006). Mechanisms of disease: neuropathic pain—a clinical perspective. Nat. Clin. Pract. Neurol..

[36] Bennett G.J., Xie Y.K. (1988). A peripheral mononeuropathy in rat that produces disorders of pain sensation like those seen in man. Pain.

[37] Kukkar A. (2013). Implications and mechanism of action of gabapentin in neuropathic pain. Arch Pharm. Res. (Seoul).

[38] Woolf C.J., Mannion R.J. (1999). Neuropathic pain: aetiology, symptoms, mechanisms, and management. Lancet.

[39] Pitchford S., Levine J.D.J.N.l. (1991). Prostaglandins sensitize nociceptors in cell culture.

[40] Sommer C., Kress M.J.N.l. (2004). Recent findings on how proinflammatory cytokines cause pain: peripheral mechanisms in inflammatory and neuropathic hyperalgesia.

[41] Woolf C. (1997). Cytokines, nerve growth factor and inflammatory hyperalgesia: the contribution of tumour necrosis factor α.

[42] Honore P. (2006). Interleukin-1αβ gene-deficient mice show reduced nociceptive sensitivity in models of inflammatory and neuropathic pain but not post-operative pain.

[43] Mittal M. (2014). Reactive oxygen species in inflammation and tissue injury. Antioxidants Redox Signal..

[44] Sun L., Kanwar Y.S. (2015). Relevance of TNF-α in the context of other inflammatory cytokines in the progression of diabetic nephropathy. Kidney Int..

[45] Mallet M.-L. (2020). The role of oxidative stress in peripheral neuropathy. J. Mol. Neurosci..

[46] Kim H.K. (2004). Reactive oxygen species (ROS) play an important role in a rat model of neuropathic pain. Pain.

[47] Hall E.D. (2016). Lipid peroxidation in brain or spinal cord mitochondria after injury. J. Bioenerg. Biomembr..

[48] Deneke S.M. (2000). Thiol-based antioxidants. Curr. Top. Cell. Regul..

[49] Kutlu T. (2009). Protective effect of dietary apricot kernel oil supplementation on cholesterol levels and antioxidant status of liver in hypercholesteremic rats. J. Food Agric. Environ..

[50] Vardi N. (2013). The protective effects of Prunus armeniaca L (apricot) against methotrexate-induced oxidative damage and apoptosis in rat kidney. J. Physiol. Biochem..

[51] Ramadan A. (2020). The pharmacological effect of apricot seeds extracts and amygdalin in experimentally induced liver damage and hepatocellular carcinoma. Journal of Herbmed Pharmacology.

[52] Abdel-Rahman M.K. (2011). Can apricot kernels fatty acids delay the atrophied hepatocytes from progression to fibrosis in dimethylnitrosamine (DMN)-induced liver injury in rats?. Lipids Health Dis..

[53] Karabulut A. (2014). Nutri-protection and mediterranean diet: bitter apricot kernel and amygdalin treatment effects on a battery of oxidative stress and apoptosis biomarkers. J Plant Physiol Pathol.

[54] Kamel G., Awad N.E., Shokry A.A. (2018). Phytochemical screening, acute toxicity, analgesic and anti-inflammatory effects of apricot seeds ethanolic extracts. Journal of Applied Veterinary Sciences.

